# Supplementary material for: Point Defects and Their Dynamic Behaviors in Silver Monolayer Intercalated between Graphene and SiC
Source: Nano Lett. 2025 Dec 31;26(2):902–9. doi: 10.1021/acs.nanolett.5c05763 (PMC12833836; doi:10.1021/acs.nanolett.5c05763)
Supplement: Supplementary file 1 [file nl5c05763_si_001.pdf]

Supporting Information

Point defects and their dynamic behaviors in silver monolayer intercalated between graphene and SiC

Van Dong Pham,[1*] Arpit Jain[2], Chengye Dong[2,3,4], Li-Syuan Lu[2], Joshua A. Robinson[2,3,4,5], Achim Trampert[1], Roman Engel-Herbert[1]

[1]*Paul-Drude-Institut für Festkörperelektronik, Leibniz-Institut im Forschungsverbund Berlin e. V., Hausvogteiplatz 5-7, 10117 Berlin, Germany*

[2]*Department of Materials Science and Engineering, The Pennsylvania State University, University Park, PA 16802, USA*

[3]*2-Dimensional Crystal Consortium, The Pennsylvania State University, University Park, PA 16802, USA*

[4]*Center for 2-Dimensional and Layered Materials, The Pennsylvania State University, University Park, PA 16802, USA*

[5]*Center for Atomically Thin Multifunctional Coatings, The Pennsylvania State University, University Park, PA 16802, USA*

[*]Corresponding author: pham@pdi-berlin.de

Table of Contents:

1. Apparent height of monolayer Ag
2. Moiré pattern formed between 2D Ag and graphene
3. Bias-dependent imaging of defects in 2D Ag
4. Tip height dependence of dI/dV over the bright defects
5. Height profile of each defect species
6. Consecutive STM images of switching dynamics in bright defects

1. Apparent height of monolayer Ag

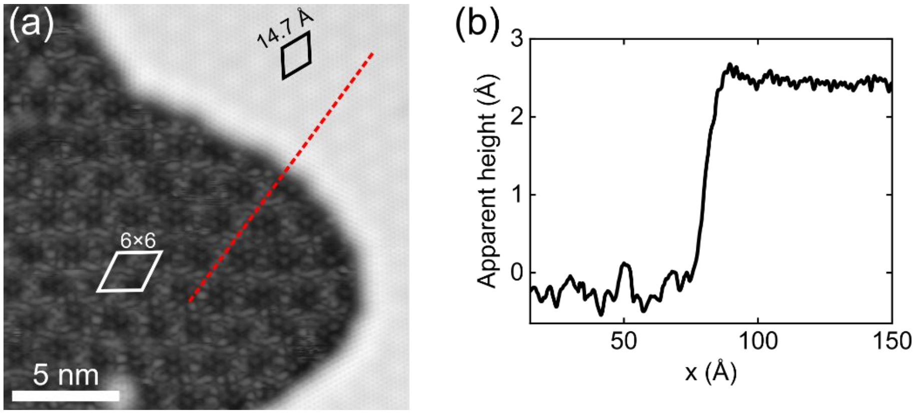

S1. (a) STM topography image of two regions between a pristine graphene/SiC (left side) and intercalated Ag (right side). The 6×6 superstructure (white rhombus) reveals a fingerprint that this area is composed of monolayer graphene on SiC without intercalants. The area on the right shows a periodicity of ~14.7 Å (black rhombus) as the result of the superposition of six graphene unit cells (2.46 Å) and five Ag unit cells (2.98 Å) in the Ag monolayer. (b) Line profile along the red

dashed line in (a), revealing a height difference between them of ~3 Å, identifying an Ag monolayer intercalated below graphene.

Figure S1 shows a transition region between a pristine graphene/SiC and intercalated Ag. The 6×6 periodicity (white rhombus), which originates from the 6√3×6√3 reconstruction on SiC, indicates no Ag intercalated between the monolayer EG and SiC in this region. In contrast, the region on the right that exhibits a less corrugated surface with a periodicity of ~14.7 Å (black rhombus) is intercalated by Ag. The apparent height difference shown in S1 (b) between the two regions is measured of ~3 Å, revealing a monolayer Ag, which is in good agreement with previous findings[1].

2. Moiré pattern formed between 2D Ag and graphene

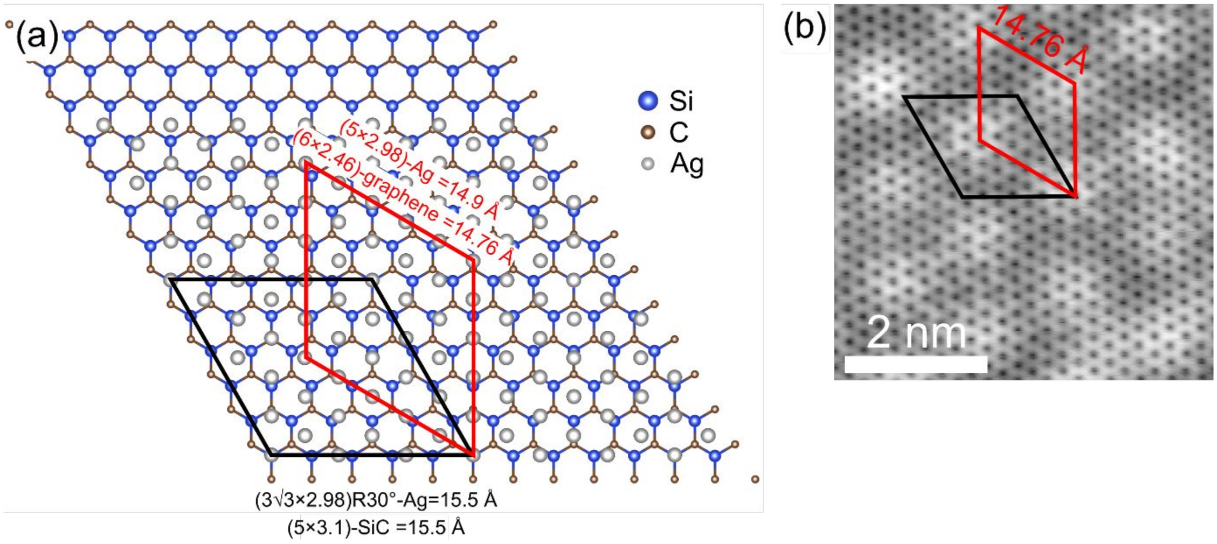

S2. (a) (3√3×3√3)R30°-Ag supercell fitting a (5×5)-SiC supercell indicated by the black rhombus[2]. The graphene layer, which is aligned with the Ag lattice, is not included for a better illustration. The moiré pattern (lattice constant of 14.7 Å, indicated by the red rhombus) observed in the experiment shown in (b), results from the superposition of six graphene unit cells (2.46 Å) and

five Ag unit cells (2.98 Å) in the Ag monolayer. Both Ag and graphene are aligned. The black rhombus supercell is not detectable by STM imaging. (b) Experimental STM topography image (0.05 V, 100 pA) of intercalated Ag exhibiting a moiré pattern with a periodicity representing the red rhombus shown in (a).

Fig. S2(a) represents the $(3\sqrt{3}\times3\sqrt{3})R30°$-Ag supercell fitting a (5×5)-SiC supercell (black rhombus). The moiré pattern, which is shown in the STM image of Fig. S2(b) [which is also shown in Fig. 1 (a) and 2(a)], results from the superposition of six graphene unit cells and five Ag unit cells (2.98 Å) of the underlying monolayer, characterized by the 14.7 Å (depicted by red rhombus in Fig. S2 (a). In Fig. S2(a), the graphene overlayer is not displayed for better clarity). This moiré pattern periodicity represents the size of the red rhombus shown in (a). Note that the graphene remains rotated by 30° with respect to the SiC lattice after intercalation. This is because the intercalation only lifts the graphene buffer layer without inducing an additional rotation, consistent with LEED measurements reported in previous studies[3,4]. From this moiré pattern periodicity (14.7 Å), we deduced an epitaxial matching between Ag and SiC, which leads to the conclusion that each $(3\sqrt{3} \times 3\sqrt{3})$-Ag supercell fits well with a (5 × 5)-SiC supercell with an Ag-Ag spacing of 2.98 Å, depicted by the black rhombus in Fig. S2 (a). Note that the black supercell was not detectable in the STM image in Fig. S2 (b).

The lattice constant of 2.98 Å in the monolayer Ag was further confirmed by LEED measurements and DFT calculations as it exhibits a most energetically favorable configuration[2]. We define this epitaxial matching (27:25)-Ag structure according to the Ag:SiC matching ratio and mentioned in the main text.

3. Bias-dependent imaging of defects in 2D Ag

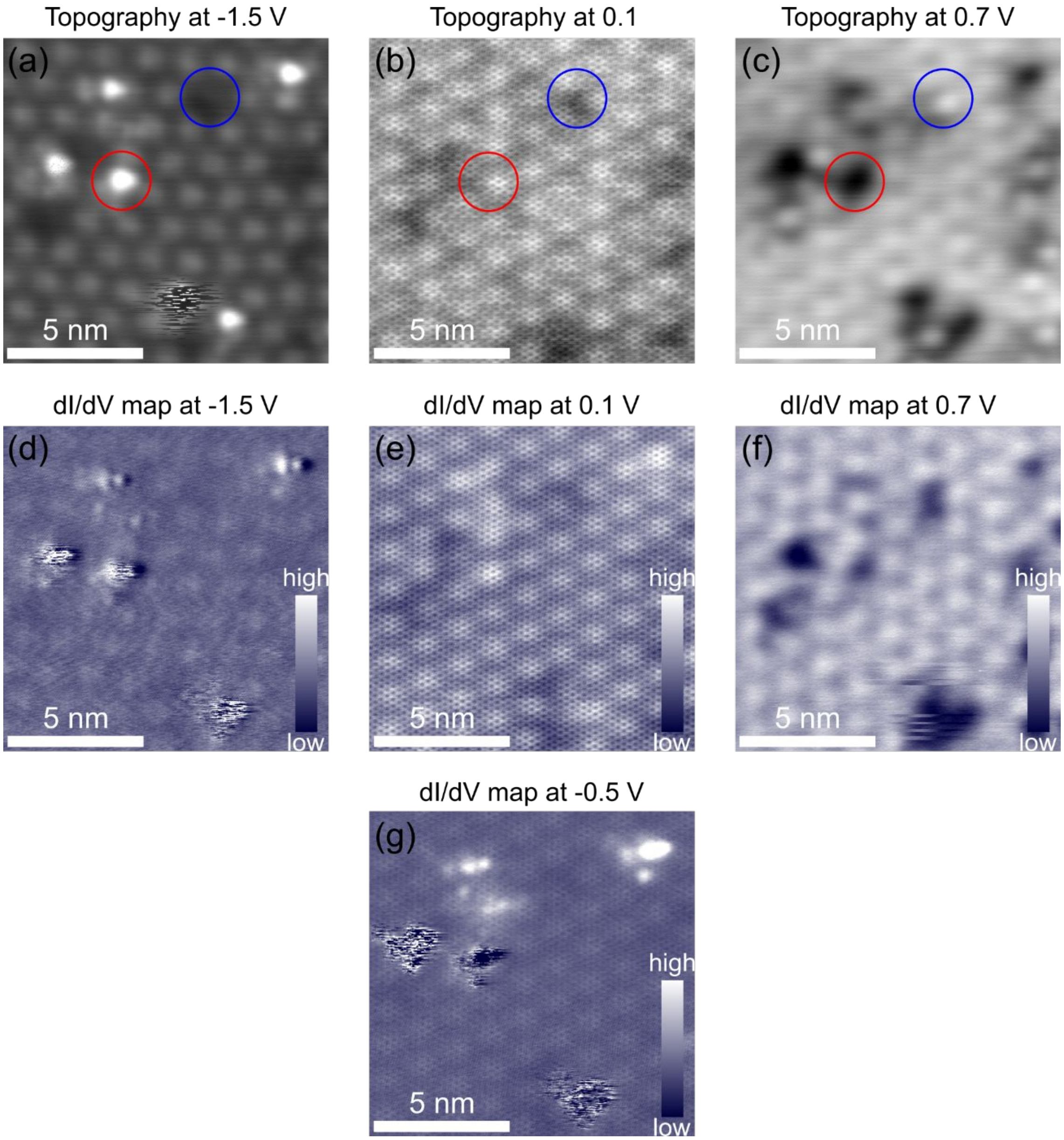

S3. (a-c) STM topography images of an intercalated Ag area taken at different bias voltages, revealing bias-dependent contrast of bright and dark defects, indicated by red and blue circles, respectively. (d-f) Corresponding dI/dV maps acquired with a constant-tip height at the same area as in (a-c), respectively. At 0.1 V, the area shows only a graphene honeycomb lattice. This indicates that the defects are located well below the graphene layer and not adsorbed on top of it.

The bias-dependent topography contrast of the defects and their dI/dV density of state maps reflects bias-dependent wavefunction overlaps of intercalated Ag and graphene with the STM tip. Note that the graphene is also visible at -0.5 V at which the defects are clearly observed in S3 (g).

The satellite spots adjacent to the bright defects, observed at -1.5 V and -0.5 V, vary from defect to defect in both brightness and number. We tentatively attribute these features to possible charge transfer from the defect to the nearby Ag atoms. Depending on the spatial extent of this charge redistribution, which may vary with local environment, one, two or several Ag atoms could be affected and become visible as small bright spots.

4. Tip height dependence of dI/dV over the bright defects

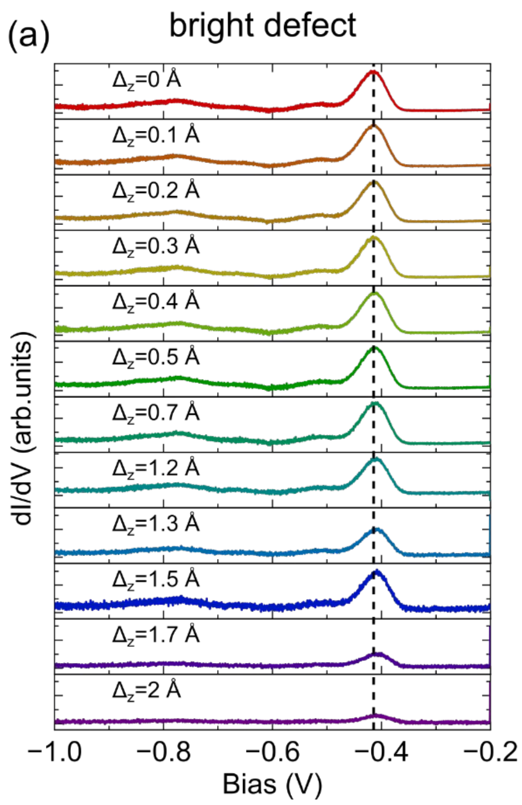

S4. Series of dI/dV spectra acquired on a bright defect (a) and on a dark defect (b) with varying tip-sample variation (Δ_z) (indicated above each spectrum at an initial tunneling condition: 0.1 V, 100 pA). Note that tip-sample variation (Δ_z) is relative to the initial tip height, defined by the set point. Δ_z=0 Å implies that the spectra were acquired without changing a z-offset and Δ_z=0.1 Å, for instance, implies that the tip is retracted 0.1 Å away from the surface. In both cases, the occupied and unoccupied peaks show almost no shift as a function of tip-sample distance. A small progressive shift over the range of ~2 Å is observed, but this is not an indication of the charging peak.

5. Height profile of each defect species

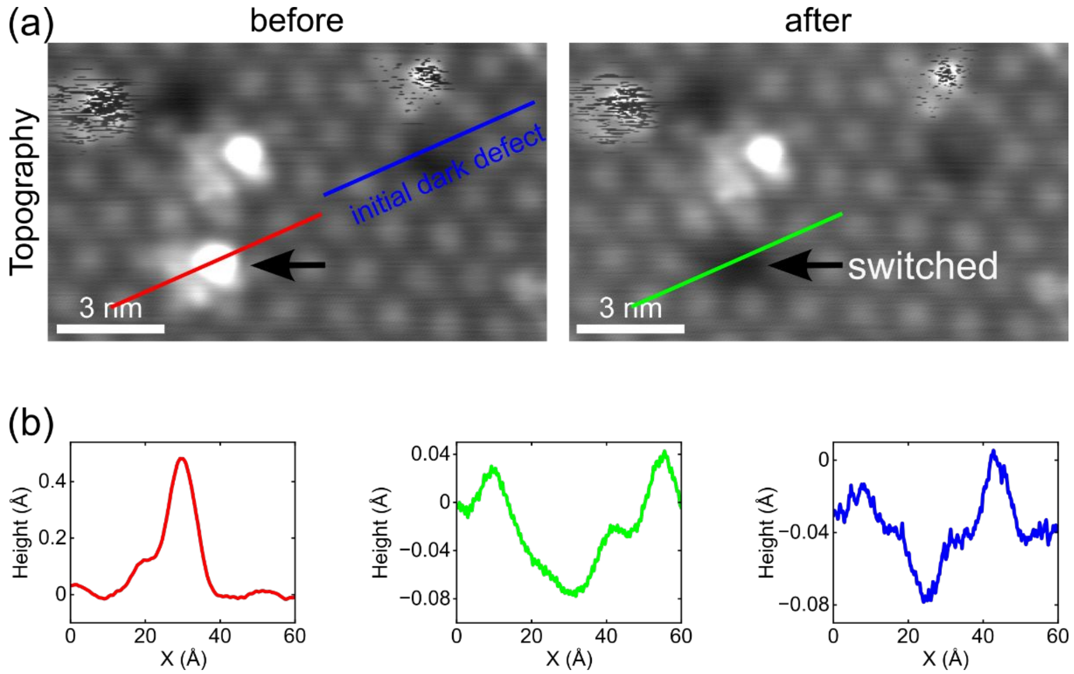

S5. (a) STM images (-0.73 V, 100 pA) of bright and dark defects before (left panel) and after (right panel) the lower bright defect switched (indicated by the black arrow). (b) Corresponding

height profiles of the lower bright defect before switching (red curve) and after switching (green curve) in comparison with the height of an initial nearby dark defect (blue curve).

6. Consecutive STM images of switching dynamics in bright defects

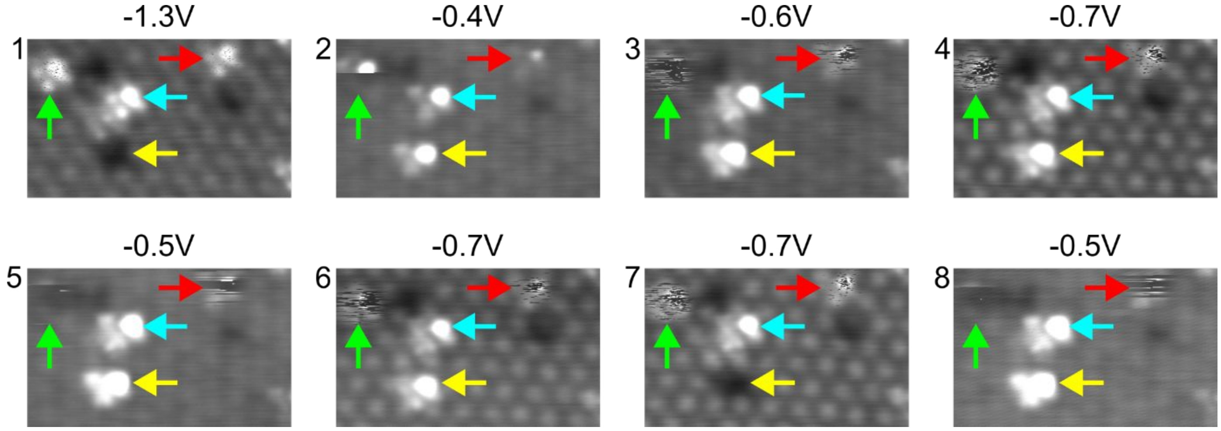

S6. Sequence of STM images (labelled 1-8) acquired at different biases over time, showing different switching behaviors of four bright defects indicated by green, red, cyan, and yellow arrows under the same tip condition. The defect marked by the cyan arrow remains stable across the image sequence, whereas three other bright defects (indicated by green, red, and yellow arrows) exhibit fast and slow switching rates. Upon switching, the defects marked by green and yellow arrows transform into dark vacant sites.

References

(1) Briggs, N.; Gebeyehu, Z. M.; Alexander, V.; Zhao, T.; Wang, K.; De La Fuente Duran, A.; Bersch, B.; Bowen, T.; L. Knappenberger, Jr., K.; A. Robinson, J. Epitaxial Graphene Intercalation: A Route to Graphene Modulation and Unique 2D Materials. *arXiv.1905.09261*. Submitted on 22 May **2019**, last revised 30 May **2019**. https://doi.org/10.48550/arXiv.1905.09261. (accessed 2025-11-10).

(2) Jain, A.; Zheng, B.; Datta, S.; Ulman, K.; Henz, J.; Wei-Jun, M.; Pham, V. D.; He, W.; Dong, C.; Lu, L.-S.; Vera, A.; Auker, W.; Wang, K.; Hengstebeck, B.; Henshaw, Z. W.; Wetherington, M.; Blades, W. H.; Knappenberger, K.; Quek, S. Y.; Starke, U.; Huang, S.; Crespi, V. H.; Robinson, A. Defect-Mediated Phase Engineering of 2D Ag at the Graphene/SiC Interface. *arXiv.2511.07151.* Submitted on 10 Nov **2025**. https://doi.org/10.48550/arXiv.2511.07151. (accessed 2025-11-15).

(3) Rosenzweig, P.; Starke, U. Large-Area Synthesis of a Semiconducting Silver Monolayer via Intercalation of Epitaxial Graphene. *Phys. Rev. B* **2020**, *101* (20), 201407. https://doi.org/10.1103/PhysRevB.101.201407.

(4) Riedl, C.; Coletti, C.; Iwasaki, T.; Zakharov, A. A.; Starke, U. Quasi-Free-Standing Epitaxial Graphene on SiC Obtained by Hydrogen Intercalation. *Phys. Rev. Lett.* **2009**, *103* (24), 246804. https://doi.org/10.1103/PhysRevLett.103.246804.
